# Collectanea

**Published:** 1827-03

**Authors:** 


### COLLECTANEA.

Floriferis ut apes in saltibus omnia libant,

Omnia nos, itidem, depascimur aurea dicta.

### PHYSIOLOGY.

*Case of Spontaneous Depilation.* By W. J. CARSON, Esq. (From the Edinburgh Journal of Medical Science.)

Mr. J. H. in B——, county of Cumberland, unmarried, ætat. thirty-five. The patient is a strong-built, muscular man, fully six feet in height, free from either local or constitutional disease, excepting that which I am about to describe. Some years ago, had one or two slight attacks of pleuritis, which were readily subdued by the common mode of treatment, without leaving any bad effects; and, with the exception of those, has always enjoyed an excellent state of health, and up to the present time been actively employed in agricultural pursuits.

He is so completely denuded of hair, that, after the minutest search, I was unable to find even the vestige of one upon any part of his head, body, or extremities, —even the supercilia, vibriscæ, and pili auriculares, have entirely disappeared; whilst his skin is of a healthy colour, and free from the smallest perceptible irregularity of surface. When examined upon the circumstances connected with the first appearance of the disease, he stated, that the hair of his head was always of a lightish cast; his beard, whiskers, &c. reddish, and that every where it was particularly plentiful and strong. This I can myself confirm, recollecting to have seen him about a year ago in his usual good state of health, when, even upon the backs of his hands, it seemed to be more abundant and stronger than in most people.

States, that he observed it first when shaving, on or about the 14th of March,

1826, in the appearance of a small bare spot over the buccinator muscle of the right cheek: and that, as he continued to shave, the hair, instead of being cut smooth with the surface, for the most part came away by the roots, and in such quantity, that, after having shaved a few more times, he had not a hair left upon his face. On the same 14th of March, he went to a person in the neighbourhood for the purpose of having his hair cut. The individual whom he employed to do so observed a small bared spot over the transverse ridge of the occiput, on the right side, which from that day forward gradually extended, till, before the middle of May, he was so totally denuded as not to have a solitary hair on any part of his frame. During the progress of the disease, he did not experience the least pain, sickness, or uneasiness, excepting what arose from the disagreeable inconvenience of the hairs falling into his victuals, &c. upon the smallest motion.

Another no less remarkable feature of the case is, that the finger-nails seem to participate of the general disease; as, though they have not actually dropped out, yet they are shrunk and withered, and appear not to receive any nourishment by their roots: at the same time they are of a whitish colour, brittle consistence, and very ragged at their extremities. This appearance did not present itself till perhaps three months after the loss of the hair; and, what is singular, only the finger-nails are thus affected, whilst those of the toes retain their natural colour and consistence.

The head perspires so freely that it is always moist, whilst there is no perceptible perspiration upon any other part. For the last three months he has worn a wig during the day, and a warm cap at night; but, whether the wig be on or off, the perspiration over the whole surface of the head is the same.

Previous to November 1825, he was accustomed to partake freely of spirituous liquors, though by no means what is generally called a drunkard; but, about that time, having taken some dislike to spirits, he all at once and entirely refrained from them: he, however, still continued to take strong ale, and perhaps even in greater quantity. In this consisted the sole difference of his regimen, or way of living from what it had always been. About the month of June last, however, some one having told him that his abstinence in this respect was the cause of the hair falling away, he again began to take spirits as before—freely, but not immoderately.

### *Case of Ulcerated Cornea, from Inanition.* By Joseph Brown, M.D. (From the Edinburgh Journal of Medical Science.)

On going yesterday to visit a poor babe, which you know I was attending at ——, I found that both corneæ had become opake over a considerable portion of their surface, and that ulceration had commenced; and, on repeating my visit today, I found that this ulceration had proceeded so rapidly, that, should the little patient live four-and-twenty hours longer, I am convinced that the contents of the eyes will be discharged. The child, which is six months' old, is as much emaciated as possible, the movement of the intestines being visible through the parietes of the abdomen. It was born prematurely, and never had the breast-milk. Since my attendance, which began ten days ago, its diet has consisted of asses' milk, sugar, and biscuit-powder: but, from the feebleness of the digestive function, it never seems to have derived sufficient nourishment from any food that has been given to it, and has been harassed ever since its birth with bowel complaints.—Compare this case with Magendie's account of the dogs fed, or rather starved, on sugar.

---

### PATHOLOGY.

*Distinction between Small-Pox and the Varioloid Disease.*—At a late meeting of the Institut Royal de France, M. Moreau de Jonnés read a Memoir, entitled "Recherches pour determiner le Caractere et les Effets de la Variole, et pour decouvrir l'Origine de cette Maladie," in which he states that the varioloid disease (*la varioloide)* differs from small-pox. Without implying any assent to his propositions, we subjoin his statements:—

The varioloid disease differs from true small-pox essentially in its effects—1st. In attacking individuals vaccinated, inoculated, and those having already had the small-pox naturally. 2d. In constantly putting on an unfavourable character, and being often fatal when it attacks individuals not vaccinated, whether they have had the small-pox naturally, or by inoculation, or not at all.

It differs also in its symptoms—1st. By the tubercular form of the pustules, which is more distinct, and common to a greater number of papulæ. 2d. By nausea and vomitings, which accompany the commencement of the disease more constantly than in ordinary small-pox. 3d. By a greater disposition to affect the lungs, to produce cough, and a sense of fulness and oppression. 4th. By the pustules not being so deep, and containing a liquid often remaining limpid instead of becoming purulent. 5th. By the crusts not crumbling into powder between the fingers, as in those of common small-pox. 6th. By the absence of fever. 7th. By the marks left, which, though indelible, are smaller and shallower, and more confined to the surface of the skin. 8th, and lastly. By a less characteristic smell than in ordinary small-pox.

The varioloid disease has a distinct existence and mode of propagation. It exists simultaneously with small-pox and chicken-pox, and may either follow or precede their irruption, or accompany them. Physicians confound it with both. Many consider it not as a species, but a variety,—a modification of the common small-pox, produced by the agency of the vaccine virus on it. The opinion of its identity with small-pox as to its origin, is supported by experiments, which it would be unnecessary to repeat. It has been stated, that inoculation with the virus from the varioloid eruption has produced ordinary small-pox. In examining the difference of symptoms and power of the two diseases, and particularly by tracing attentively the progress of the varioloid disease, he is inclined to think, with some of the practitioners of America and the North of Europe, that it is a new species.

If it is true, as they say, that the degeneration of the vaccine virus is the cause of the increase of small-pox, and that the mortality which happens by its increase is the effect of that degeneration, without doubt there would be no difference in countries where that practice has been simultaneously adopted. Vaccination would be no more a preservative in Bohemia and Russia, than in England, Scotland, in a part of France, and the United States. Now, this is not the case. In London, in 1817, the mortality caused by small-pox was 1 in 19; at Paris, in 1825, 1 in 18; whilst at Prague, in 1810, it was 1 in 265, and of nearly 100,000 inhabitants, the small-pox carried off only 14 people. In Sweden, even in 1824, its ravages have been still less, and the efficacy of vaccination had not there experienced any diminution till the appearance of the varioloid disease in that country, which, in 1779, lost 15,000 individuals in a population of 1,958,000; which made a mortality of 133 inhabitants by the small-pox. In

1822, there died but 11 out of 2,697,000, or 1 in 2,045. But a mischievous invasion of the varioloid disease has altered this happy state of things. At those same times, the same disease had caused a mortality in London of 1 in 1000; and at Paris of 1 in 540. So great a mortality from small-pox shows that small-pox is resuming in these two capitals its epidemic character, which it had lost for thirty years, since the introduction of vaccination. The small mortality by this disease in Bohemia and Persia shows, on the contrary, in proving its existence, that it is powerless, and cannot surmount the obstacles opposed to it by vaccination.

We must then look for some other cause for the increase of small-pox contagion, than the weakening of the protecting influence of vaccination, since the increase is only in some countries, excluding many others. It will be found that those countries where the varioloid disease has not yet appeared are in the eastern part of Europe, far from the shores of that continent. Following this indication, one is soon convinced that the maritime countries of both hemispheres have been the first attacked: in Europe, Great Britain, the Hanse Towns, and France; in America, the United States. In these countries, the towns where commerce is most active and most extended, have been first visited by this scourge: London, Edinburgh, Hamburgh, Paris, New York, and Philadelphia, are the places which have first felt its effects.

The same observations may be made on the two shores of the Atlantic Ocean: it is in the ports most frequented that this new species of small-pox has first appeared; and the people who have first felt its effects are those whose ships almost exclusively navigate the Indian Ocean. It is in the United States and Great Britain that it has first shed its fatal influence, and just at the time when the ships of these two countries redoubled their efforts to increase their commercial relations with eastern countries.

The author goes on to state, that, when England first pushed her conquests in India, there appeared in the English ports a species of distinct small-pox, observed by Dr. Mead, and described by him under the name of Variola Siliquosa. There is no doubt that, from time immemorial, there has existed in Hindostan several kinds of varioloid diseases, and it is extremely probable that those known to Europeans took their origin in that region of Asia. It appears (he continues) that the vaccine virus exists in many parts of Asia, and that its properties have been recognised there for a long time. Among some of the wandering tribes inhabiting Persia vaccination is known, and frequently propagated; and, what is extraordinary, it is more frequently from sheep than cows that the virus is communicated to the shepherds. In Persia, the sheep are milked as much as cows are with us. In considering the frightful mortality of small-pox in Asia, one is disposed to believe that among their species there exists not only the small-pox commonly known in this country since the tenth century, but also this kind recently imported, and designated by the name of Varioloid.

M. Jonnes sums up with the following conclusions:

1st. That the varioloid eruption is a species of small-pox, distinct in its symptoms, effects, and origin, from that introduced into Europe eight centuries ago.

2d. That there is reason to believe that this new species belongs in the first instance (as was the case with the old,) to the tropical regions of Asia, whence it has been imported to the United States and England, within the last ten years.

3d. That it is only since vaccination has begun to show itself a less certain protector, that the varioloid disease has appeared in America and Europe, and has there been propagated, first by maritime communications, and then by degrees spreading inland.

4th. That this species, which seems analogous to the Variola Siliquosa of Dr. Mead, the appearance of which in England coincided with the first conquests of the English in India, is more dangerous than common small-pox when not modified, and causes a greater mortality.

5th. That neither the ordinary small-pox in the natural way, nor communicated by inoculation, nor the vaccine virus, protest against it. protec

6th. That sometimes the vaccine virus weakens and modifies its pernicious influence; that, in the United States, of fifty individuals vaccinated and seized with this new species, none died; while, of one hundred not vaccinated who were attacked, one half perished. Whence it follows that, although vaccination does not secure from this scourge, it so modifies it as to blunt its virulence, and therefore is a most useful and indispensable precaution.—*Revue Medicale.*

